# BMPR1B Up-Regulation via a miRNA Binding Site Variation Defines Endometriosis Susceptibility and CA125 Levels

**DOI:** 10.1371/journal.pone.0080630

**Published:** 2013-12-05

**Authors:** Cherry Yin-Yi Chang, Yi Chen, Ming-Tsung Lai, Hui-Wen Chang, Jack Cheng, Carmen Chan, Chih-Mei Chen, Shan-Chih Lee, Ying-Ju Lin, Lei Wan, Pei-Wen Tsai, Su-Han Yang, Ching Chung, Jim Jinn-Chyuan Sheu, Fuu-Jen Tsai

**Affiliations:** 1 Department of Obstetrics and Gynecology, China Medical University Hospital, Taichung, Taiwan; 2 Department of Public Health, China Medical University, Taichung, Taiwan; 3 School of Medicine, China Medical University, Taichung, Taiwan; 4 Human Genetic Center, China Medical University Hospital, Taichung, Taiwan; 5 Department of Pathology, Chung Shan Medical University Hospital, Taichung, Taiwan; 6 School of Medicine, Chung Shan Medical University, Taichung, Taiwan; 7 College of Medical Science and Technology, Chung Shan Medical University, Taichung, Taiwan; 8 School of Chinese Medicine, China Medical University, Taichung, Taiwan; 9 Department of Health and Nutrition Biotechnology, Asia University, Taichung, Taiwan; 10 School of Post-Baccalaureate Chinese Medicine, China Medical University, Taichung, Taiwan; Baylor College of Medicine, United States of America

## Abstract

**Background:**

Bone morphogenetic protein receptor I B (BMPR1B) is a transmembrane receptor mediating TGF-β signal transduction. Recent studies indicate a tumor suppressor role for BMPR1B in ovarian cancer. Polymorphism at *BMPR1B* 3′UTR within the miR-125b binding site alters its binding affinity toward the miRNA, which may result in insufficient post-transcriptional repression.

**Methods:**

Single-nucleotide polymorphisms rs1970801, rs1434536, and rs11097457 near the miR-125b binding site in *BMPR1B* were genotyped by *Taqman* assay on 193 endometriosis patients and 202 healthy controls. BMPR1B and CA125 levels in ectopic endometrial tissues were evaluated by quantitative PCR and immunohistochemistry. Luciferase reporter assay was utilized to verify regulatory roles of *BMPR1B* 3′UTR with allelic variants of rs1434536 in a cell line model. Cell proliferation and migration were recorded, while expression of BMPR1B, CA125, glucocorticoid receptor (GCCR) and IL-1β were measured by quantitative PCR in endometrial cells transfected with wild-type or mutated miR-125b.

**Results:**

This study found two endometriosis-associated SNPs, rs1434536 (*P* = 0.010) and rs1970801 (*P* = 0.0087), located within and next to a miR-125b binding site on *BMPR1B*. Interestingly, patients with homozygous variant alleles at rs1434536 showed significantly lower serum CA125 levels. Immunohistochemistry staining further confirmed inverse correlation between BMPR1B and CA125 levels in three rs1434536 genotypes. Cell assays demonstrated the variant allele of rs1434536 up-regulating BMPR1B at both mRNA and protein levels, which negatively correlated with CA125 and IL-1β levels. Disruption of the binding between miR-125b and BMPR1B hampered abnormal cell proliferation.

**Conclusions:**

SNPs of *BMPR1B* within and next to the miR-125b binding site manifested strong correlation with endometriosis development in a Taiwanese cohort. Disrupting the binding of miR-125b toward *BMPR1B* would increase protein expression, diminishing abnormal cell proliferation as well as serum and cellular CA125 levels. Genetic variation at the miR-125b binding site may play functional roles to protect against endometriosis progression.

## Background

Endometriosis is a benign yet debilitating gynecological disease associated with chronic pelvic pain, dysmenorrhea, and infertility. This common reproductive disorder, characterized by presence and growth of endometrium-like tissues outside the uterine cavity, affects approximately 10% of reproductive age women [1]–[3]. Overall, individual susceptibility is influenced by multiple factors, such as hormone aberration, abnormal immune response, environment, individual anatomy, genetic or epigenetic predisposition [1], [4]–[6]. Though several theories are proposed regarding the etiology, molecular mechanisms contributing to pathogenesis remain unclear.

MicroRNAs (miRNAs) are small non-coding single-stranded RNAs of 20–24 nucleotides that regulate gene expression at transcriptional and post-transcriptional levels via base-pairing with complementary sequences within mRNA molecules [7]. As an essential component of epigenetics, miRNAs participate in a gamut of biological processes: cell differentiation, proliferation and/or apoptosis. Recent studies discovered differential expression of miRNAs in endometriosis tissues as compared to normal endometrium, indicating that epigenetic regulation by miRNAs plays important roles in endometriosis development [8], [9]. Targeting genes by miRNA binding accelerates target degradation or translational repression, depending on complimentary degree between target sites and miRNAs [10]–[12]. Single-nucleotide polymorphisms (SNPs) within miRNA binding sites can hinder target gene recognition, augmenting target gene expression. Thus, SNPs within or near a miRNA target site may genetically predispose to endometriosis as an epigenetic modulator, which may explain notions from previous familial and twin studies suggesting endometriosis as inherited in a polygenic/multifactorial manner [13]–[15].

BMPR1B, a member of the bone morphogenic protein (BMP) receptor family of transmembrane serine/threonine kinase, belongs to the transforming growth factor-β (TGF-β) superfamily [16], whose members are dynamically expressed in the endometrium during menstruation, pregnancy, and endometriosis [17]–[19]. TGF-β level rose about ten times higher in peritoneal fluid from endometriosis patients compared to that from normal women [20]. TGF-β was later proven to be essential for maintaining integrity of ECM, preventing breakdown of endometrial tissue, promoting angiogenesis, and enhancing invasiveness of endometriotic cells [19], [21], [22]. Similar to common TGF-β signal transduction, BMP signaling is mediated by serine/threonine kinase receptors BMPRI and BMPRII, which was originally discovered as a developmental inducer of cartilage and bone formation [23]–[25]. Recent studies indicate that BMP signaling is a key regulator during embryo, heart, and neural development [26]. Significantly, BMP members and their receptors were proven crucial to development and normal reproductive function of ovaries, while acting as tumor-suppressors against ovarian cancer [27].

To elucidate the association of *BMPR1B* with endometriosis, we performed genetic and functional study of SNPs within and next to the mir-125b binding site on *BMPR1B*, previously identified as genetic risk factors of estrogen receptor-stratified breast tumors in a genome-wide association study [28]. Recent studies also identified mir-125b as one of several miRNAs differentially expressed in endometriosis that may regulate genes involved in cell proliferation, e.g. ERBB2/3, TNF, and TP53 [8]. Polymorphism of the miRNA binding site appeared to negate miR-125b directed repression and enhance BMPR1B production. Our study of BMPR1B in endometriosis may help understanding of disease etiology and provide new perspectives on prognosis and therapeutic approaches.

## Methods

### Study population

A total of 193 pathology-proven endometriosis tissues were collected at China Medical University Hospital from 1998 to 2010. Study subjects were diagnosed with ovarian cyst by ultrasound, and ectopic endometrial tissues were collected during laparoscopic procedure. Lesions which may present endometriosis were either excised or electro-cauterized during operation and sent for pathology. Subjects without pathology-proven endometriosis were excluded. Clinical symptoms related to endometriosis also surfaced, including dysmenorrhea, lower abdominal pain, infertility or menorrhagia. Patients were classified into different endometriosis stages according to the American Society of Reproductive Medicine [29]: Stage 1, minimal (2.6% of total patients); Stage 2, mild (16.6%); Stage 3, moderate (30.5%); Stage 4, severe (50.3%). However, their menstrual stages at the time of surgery were not recorded. Control group consisted of 202 women with matching age profiles certified as healthy by regular physiological check-ups. This study was approved by the Institutional Review Board at China Medical University Hospital with written consent from each participant.

### Genotyping of single nucleotide polymorphisms

Peripheral blood was collected and placed in EDTA-containing tubes. Genomic DNA was extracted from peripheral blood leukocytes according to standard protocols (Genomic DNA kit; Qiagen, Valencia, CA). DNA fragments containing SNP sites were amplified by PCR, using *Taqman* SNP genotyping assay (Applied Biosystems Inc., Carlsbad, CA). All probes were designed by manufacturer, and their IDs are listed in Table S1. Two variants were tagged with distinct fluorescent dyes. Perfect match between a probe and tested DNA fragment will initiate PCR and generate positive fluorescent signal. Genotypes were detected by reading fluorescent signals of PCR products.

### Statistical analysis

Allelic frequency and genotype frequency distributions for three SNPs of patients and controls were calculated by χ^2^ analysis with SPSS software (version 10.0, SPSS Inc. Chicago, IL). A *p* value below 0.05 was considered statistically significant. Odd ratios (ORs) were derived from allelic and genotype frequency with 95% confidence interval (95% CI). Boxplots of CA125 levels in three rs1434536 genotype groups were generated by R software (http://www.R-project.org). The *p* value comparing distribution of CA125 level in three patient groups was calculated by one-way analysis of variance (ANOVA). Notches giving approximately 95% confidence intervals of medians were calculated via asymptotic normality distribution of medians [30].

### Haplotype and linkage disequilibrium analysis of SNPs

Haplotypes were inferred from unphased genotype data, using Bayesian statistical method available in Phase 2.1 software [31], [32]. SNPs in *BMPR1B* gene, rs1970801, rs1434536, and rs11097457, were analyzed in Phase 2.1. Association with clinical phenotype of endometriosis was calculated for genetic combination of two or three adjacent SNPs. The linkage disequilibrium (LD) analyses of rs1970801, rs1434536, and rs11097457 were calculated by JLIN software (ver. 1.60; http://www.genepi.org.au/jlin, Western Australian Institute for Medical Research), and pair-wise LD maps constructed for both patient and control groups [33].

### Immunohistochemistry

Paraffin sections of ectopic endometrial tissues were deparaffinized in three changes of xylenes, then rehydrated, and subsequently immersed in 10 mM sodium citrate buffer (pH 6.0) and heated in a 95°C water bath for 20 minutes to retrieve antigens. After cooling, sections were incubated with PBS for 60 minutes, followed by anti-BMPR1B antibody (Sigma-Aldrich), anti-CA125 antibody (Sigma-Aldrich) and anti-CD10 antibody (Sigma-Aldrich) at 1∶50 dilution overnight at 4°C. After rinsing, sections were revealed by EnVision Detection System (Dako, Denmark) and counterstained with hematoxylin (Sigma-Aldrich). Because blood contamination in samples made it improper to compare staining patterns directly among different genotypes, we performed correlative studies on serial sections usng anti-CD10 staining as the internal control to adjust immunointensity. Immunostaining was independently scored by two pathologists and labeled as undetectable, weak staining, or strong staining. All biopsy samples were from ectopic endometrium.

### Reporter assay

A 300bp PCR product that contains the miR-125b binding site in *BMPR1B* 3′UTR [28] was cloned into pGL4.73 vector (Promega; Madison, WI) replacing the SV40 late poly(A) region between cutting sites XbaI and BamH1 (Table S2). Plasmids containing either C or T allele of rs1434536 were designated pGL4.73-C or pGL4.73-T, and then sequenced to confirm their identity. The pGL4.73 vector without insertion served as positive control. Endometrial cancer cell lines, HEC-1-A and RL95-2, purchased from Bioresource Collection and Research Center (BCRC), Food Industry Research and Development Institute (FIRDI), Taiwan, were plated in 24-well plates, then transfected with pGL4.73 (-C, -T or empty vector). Cells were co-transfected with 0.5 μg pTAL-SEAP (Takara Bio Inc., Japan) per well for normalization of transfection efficiency. At 48 hrs post-transfection, luciferase activities were measured and normalized according to alkaline phosphatase activities. Results were average values of four parallel experiments and plotted as percentage change of luciferase activity compared to controls.

### Cell culture, transfection and sorting

Plasmid vector pCMV-MIR (Origene, Rockville, MD) with green fluorescein protein (GFP) reporter was used to construct miR-125b-wt plasmid. A guanine-to-adenine mutation was introduced to the miR-125b plasmid at the site complementary to rs1434536, generating MIR-125b-mt, using QuikChange II site-directed mutagenesis kit (Agilent Technologies, Inc.; Santa Clara, CA). The resultant vector was verified by sequence analysis. For transfection analysis, 5×10^6^ HEC-1-A cells were seeded in 6-cm dish and transfected with each plasmid, using Lipotectamine system (Invitrogen), as per manufacturer's protocol. G418 (Sigma-Aldrich, St. Louis, MO) was added to culture medium at 24 hrs post-transfection to enrich positively transfected cells. At 48 hrs post-transfection, cells were detached, washed, and resuspended in PBS and sorted by their GFP levels via flow-cytometry (Becton Dickinson, San Jose, CA), retaining over 90% positively transfected cells. Transfection efficiency was rechecked by counting fluorescent cells under microscope before and after sorting.

### Growth and migration assay

To measure cell growth, HEC-1-A cells which had higher transfection efficiency were transfected with vectors containing either GFP, miR-125b-wt or miR125b-mt. Cells were plated at 48 h post-transfection and maintained for five days with G418-containing medium to enrich transfected cells. At different time points, cells were counted using hemocytometer after washing away dead cells with culture medium. Data represent mean values (± s.d.) of four independent measurements. For migration assay, equal numbers (3×10^4^) of cells were seeded into a Radius™ 24-Well Cell Migration Assay (Cell Biolabs Inc., San Diego, CA). After the chambers were incubated for 24 hrs, center gels were removed to allow migration monitoring for 48 hrs.

### Quantitative reverse transcriptase-PCR

For quantitative reverse transcription PCR analysis (Q-PCR), ectopic endometrial tissues or sorted cells were collected for RNA extraction using RNeasy Mini-Kit (Qiagen; Courtaboeuf, France). RNA concentrations were determined by NanoDrop ND-1000 spectrometer (Thermo Scientific, USA) and RNA integrity was assessed with Bioanalyzer 2100 (Agilent Technologies; Massy, France). Quantitative reverse transcriptase-PCR (Q-PCR) for mRNAs of interest was performed on ViiA 7 Real-Time PCR System (ABI). Total RNA (∼800 ng) was subjected to complementary DNA synthesis and subsequently amplified during 45 PCR cycles (denature: 10 minutes at 95°C; cycles: 10 seconds at 95°C, 10 seconds at 58°C, 6 seconds at 72°C) by Power SYBR Green PCR Master Mix (ABI). Relative gene expression was evaluated in each sample as ratio of miRNA targets to glyceraldehyde 3-phosphate dehydrogenase (GAPDH) mRNA copy number to normalize mRNA expression for differences in RNA input. Primer sequences are shown in Table S2.

## Results

### SNPs in *BMPR1B* associates with endometriosis

**Figure 1:**
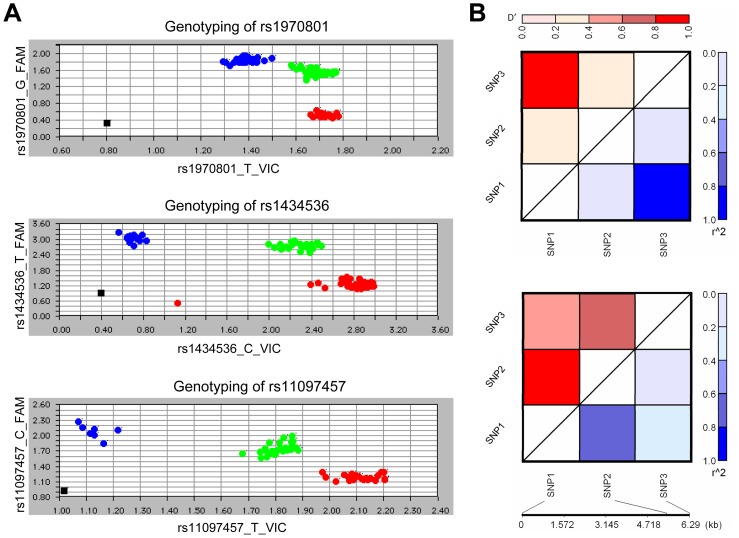
Genotyping and pair-wise LD measures for SNPs in *BMPR1B*. Genotyping was performed by *Taqman* method using DNA extracted from peripheral blood leukocytes. A. Representative fluorescent signal plots from genotyping of SNPs in *BMPR1B*. B. Through linkage disequilibrium evaluation, *D*' and *r^2^* values were plotted at top right and bottom left triangles, respectively, for normal controls (upper panel) and endometriosis patients (lower panel). Scales beneath the charts show the relative location of each SNP on chromosome 4. SNP1: rs1970801; SNP2: rs1434536; SNP3: rs11097457.

A miRNA target site variation rs1434536 resides within a partial complementary binding site to mir-125b seed region, 3′UTR of *BMPR1B*, conveying physiological effects of miR-125b [28]. Using *Taqman* assay, we genotyped rs1434536 and the two nearest SNPs in 193 endometriosis patients and 202 controls (Fig. 1A). Association analysis indicated that the variant T allele of rs1434536, which disrupts miR-125b recognition, is significantly less prevalent among patients (*P* = 0.010; OR, 0.68; 95% CI, 0.50–0.91) (Table 1). Genotype frequencies of rs1434536 also display significant difference between patients and controls (*P* = 0.031) (Table 1). Likewise, odds ratio of TT genotype indicates this homozygous variant effectively lowering susceptibility to this disease (OR, 0.43; 95% CI, 0.23–0.81) (Table 1).

**Table 1:** Genotype and allele distributions of SNPs surrounding miRNA target site of *BMPR1B* in endometriosis patients and controls.

SNP	Genotype/allele	No. (%) of patients	No. (%) of controls	*p*-value^a^	OR (95% CI)
Rs1970801	TT	29 (15.1)	43 (21.8)	0.0087	0.47 (0.27–0.82)
	GT	75 (39.1)	93 (47.2)		0.56 (0.36–0.87)
	GG	88 (45.8)	61 (31.0)		1.00
	T	133 (34.6)	179 (45.4)	0.0021	0.64 (0.48–0.85)
	G	251 (65.4)	215 (54.6)		1.00
Rs1434536	TT	18 (9.3)	35 (17.8)	0.031	0.43(0.23–0.81)
	CT	80 (41.5)	83 (42.1)		0.80 (0.52–1.23)
	CC	95 (49.2)	79 (40.1)		1.00
	T	117 (30.1)	153 (38.8)	0.010	0.68 (0.50–0.91)
	C	265 (69.9)	241 (61.2)		1.00
Rs11097457	CC	35 (18.5)	32 (16.5)	0.81	1.09 (0.62–1.92)
	CT	87 (46.0)	95 (49.0)		0.92 (0.59–1.43)
	TT	67 (35.4)	67 (34.5)		1.00
	C	157 (41.5)	159 (41.0)	0.88	1.02 (0.77–1.36)
	T	221 (58.5)	229 (59.0)		1.00

a Genotype and allele distributions were analyzed by χ^2^ test.

Abbreviations: SNP, single-nucleotide polymorphism; OR, odds ratio; 95% CI, 95% confidence interval.

The SNP rs1970801, located in an intron region of *BMPR1B* upstream from rs1434536, also proved statistically significant. While genotype distribution demonstrated homozygous TT of rs1970801 as significantly less prevalent in patients compared to controls (*P* = 0.0087; OR, 0.47; 95% CI, 0.27–0.82), allelic variation analysis also indicated T allele of rs1970801 as a protective factor against endometriosis (*P* = 0.0021; OR, 0.64; 95% CI, 0.48–0.85) (Table 1). No significant association, of allelic or genotype analyses, was found for rs11097457 in patients.

### MiR-125b binding site polymorphism and clinical phenotypes of endometriosis

We next asked whether genetic variations of these disease-associated SNPs could correlate with endometriosis-related symptoms. Grouping patients by CA125 level, we found variant T allele of rs1434536 significantly less prevalent in patients with CA125 level over the standard 35 units/ml (*P* = 0.036) (Table 2). Homozygous TT genotype also appeared to have a protective effect against abnormal increase of CA125 (OR  = 0.10, 95% CI: 0.01–0.79) (Table 2). Still, none of the SNPs demonstrated statistically significant association with any other endometriosis-related phenotypes, including infertility, pain score, and advanced stage (data not shown).

**Table 2:** Association between miR-125 target site SNP rs1434536 and CA125 levels of endometriosis patients.

Genotype/ allele	CA125>35 No. (%)	CA125<35 No. (%)	*p*-value^a^	OR (95% CI)
TT	1 (2.1)	12 (16.7)	0.036	0.10 (0.01–0.79)
CT	21 (43.8)	30 (41.7)		0.88 (0.38–1.74)
CC	26 (54.2)	30 (41.7)		1.00
T	23 (24.0)	54 (37.5)	0.028	0.53 (0.29–0.94)
C	73 (76.0)	90 (62.5)		1.00

a Genotype and allele distributions were analyzed by χ^2^ test.

### *BMPR1B* haplotypes link defined endometriosis risks and patient CA125 levels

Haplotype frequencies were analyzed for two disease-related SNPs within *BMPR1B* to confirm participation of this gene in endometriosis pathogenesis. The most common haplotype of rs1970801 and rs1434536 (G-C) was found correlated with higher risk of endometriosis (*P* = 8.7×10^-5^) (Table 3). Meanwhile, T-T haplotype consisted of both protective alleles of rs1970801 and rs1434536, portending significantly lower risks of endometriosis (*P* = 5.9×10^−3^) and serum CA125 levels (*P* = 3.7×10^−3^) (Table 3). All SNPs in this study were analyzed for linkage disequilibrium (LD), using software JLIN for patients and controls (Fig. 1B). The maps revealed distinctive linkage among three SNPs: both *D*' and *r* values indicated rs1434536 and rs1970801 as more closely linked in patients. Such LD map suggested a more rigid genetic pattern at rs1970801 and rs1434536 of *BMPR1B* preserved through generations in endometriosis patients.

**Table 3:** Association between haplotypes of rs1970801 and rs1434536 in *BMPR1B* with endometriosis and CA125 level in patients.

Haplotype	Endometriosis			CA125		
	Case (%)	Control (%)	*p*-value^a^	>35 (%)	<35 (%)	*p*-value^a^
G-C	65.0	51.2	8.7×10^−5^	64.3	52.3	0.065
T-T	21.6	28.9	5.9×10^−3^	23.2	41.4	3.7×10^−3^*
T-C	10.0	13.7	0.11	10.1	4.4	0.074
G-T	3.4	6.2	0.068	2.3	1.9	0.82

a Genotype and allele distributions were analyzed by χ^2^ test.

### Inverse correlation between BMPR1B and CA125 levels present *in vivo*

**Figure 2:**
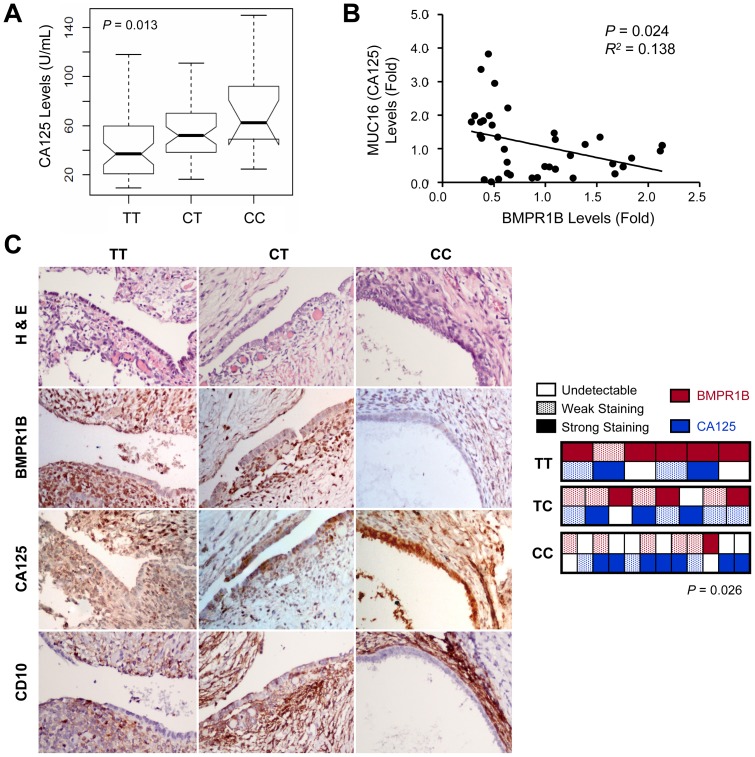
Genotypic variants presented at miR-125b binding site correlated with varied CA125 levels in patients. A. Serum CA125 levels in endometriosis patients were grouped by their rs1434536 genotypes. Bold lines indicate median values of CA125 levels in each genotype group. Notch regions define 95% confidence interval estimations of median values. B. Gene expression levels of BMPR1B and CA125 (MUC16) in ectopic endometrial tissues were measured by quantitative PCR for correlation study. C. Immunohistochemistry (IHC) staining was performed on ectopic endometrial tissues from patients. Anti-CD10 staining, a stroma cell-specific marker, serves as the reference to normalize immunointensity. Ectopic endometrium samples represented patients with rs1434536 genotype of CC, CT; and TT, as labeled on top of each column. A total of 6, 8 and 12 samples from tissue microarrays could be defined as TT, CT and CC genotype groups, respectively, and their expression levels for BMPR1B and CA125 were scored as described in method section (right panel). Box plot and ANOVA *P* value calculation were performed in R software suite.

Among clinical features, serum CA125 levels showed correlation with rs1434536 genotypes in patients. By one-way ANOVA analysis, patients with CC genotype have highest average serum CA125 level, while lowest average level was found in patients with TT genotype among three genotype groups (*P* = 0.013) (Fig. 2A). To learn whether BMPR1B expression negatively correlate CA125 levels in endometriosis patients, expression levels of both genes in ectopic endometrial tissues were measured by quantitative PCR. Results in Fig. 2B support our hypothesis that BMPR1B and CA125 expression inversely correlated in clinical samples (*P* = 0.024; *R^2^* = 0.138).

To verify our findings, we detected protein levels of BMPR1B and CA125 by immunohistochemistry using tissue microarray that contain ectopic endometrial tissues collected during laparoscopic procedure (Fig. 2C). H&E staining was performed to define histological patterns in tissues. Anti-CD10 staining, a stroma cell-specific marker, serves as the internal control to normalize immunointensity. In patients with homozygous reference alleles, with preferable miR-125b binding activity, we observed the weakest BMPR1B staining in both epithelial and stroma cells, whereas CA125 was highly expressed in epithelial cells. Budding vesicles were also observed along the edge, which may hint active production and releasing of CA125 by epithelial cells into surrounding tissue. In the heterozygous sample, moderate BMPR1B expression was detected, while cytoplasmic expression of CA125 was moderately prevalent in epithelial cells. Within samples with homozygous variant allele, more abundant BMPR1B appeared in endometriosis tissues, while the corresponding CA125 expression profoundly diminished in both epithelium and stroma regions. The staining results support our hypothesis that polymorphisms at the miR-125b binding site regulate BMPR1B levels in patients, which may determine CA125 production in endometriotic epithelial cells. Endometriosis epithelium and stroma cells may possess opposite innate efficacy to express BMPR1B and CA125.

### Rs1434536 in *BMPR1B* 3′UTR modulates miR-125b-directed gene repression

**Figure 3:**
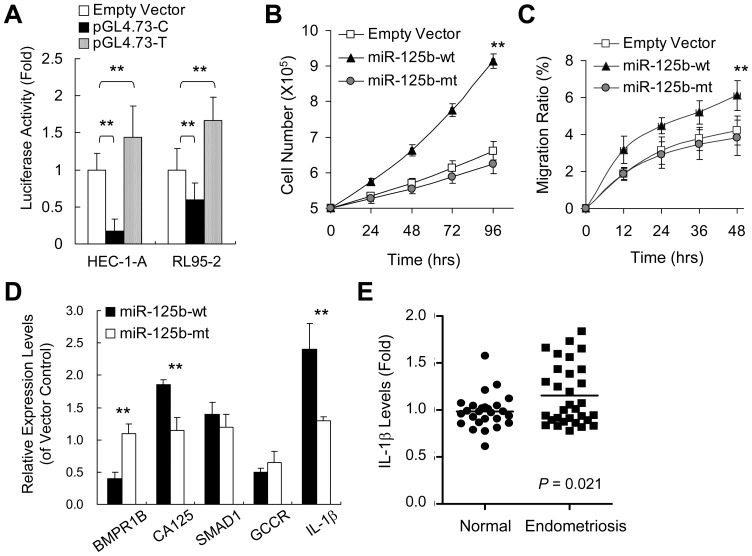
Variation disrupting recognition between miR-125b and 3′UTR of *BMPR1B* increases BMPR1B expression and inhibits abnormal cell growth *in vitro*. A. Luciferase reporter assay was employed to evaluate repression of BMPRIB in endometrial cell lines with C (HEC-1-A) or T (RL95-2) allele at rs1434536. Luciferase reporter pGL4.73 vectors, containing the reference C or variant T bearing sequence of BMPR1B 3′UTR, were transiently introduced into both cell lines. At 48h post-transfection, luciferase activities were measured and normalized to phosphatase activities. B. Cell growth of HEC-1-A cells transfected with empty vector or vector containing miR-125b-wt or miR-125b-mt with a mutated base complementary to the minor allele at rs1434536. At different time intervals, cells were counted using hemocytometer. C. HEC-1-A cells transfected with vectors containing miR-125b-wt or miR-125b-mt were counted and rated with reference to total positively transfected (GFP+) cells. D. Expression levels of known BMPR1B responsive genes were analyzed in HEC-1 cells transfected with miR-125b-wt or miR-125-mt constructs, using empty vector-treated cells as references. E. Expression levels of IL-1β in clinical samples were measured by quantitative PCR. Statistical significance is calculated by Student t-test: *, *P*<0.05; **, *P*<0.01.

To verify functional impact of rs1434536 polymorphism on miR-12b directed repression on BMPR1B level, we cloned *BMPR1B* 3′UTR with each variant allele at rs1434536 position into luciferase reporter vector pGL4.73, and then compared normalized luciferase activity among allelic variants. Figure 3A shows vectors containing *BMPR1B* 3′UTR with C-allele gave ∼83% and 41% reduction of luciferase activity in HEC-1-A and RL95-2 endometrial cells relative to empty vector, respectively. Vectors with T-allele gave ∼43% and 66% increase of luciferase activity in HEC-1-A and RL95-2 relative to empty vector, respectively. Collectively, upstream luciferase expression can be altered by 3∼10 fold via allelic variants at the miR-125b target site within *BMPR1B* 3′UTR. Our *in vitro* data indicate the possibility that binding-site polymorphism for miR-125b plays a regulatory role to control BMPR1B expression during endometriosis development.

### Complementary mutation to rs1434536 within miR-125b attenuates abnormal cell proliferation and migration

To test functional significance of binding site polymorphism, we transfected endometrial cell line HEC-1-A with plasmids containing reference (miR-125b-wt) or variant (miR-125b-mt) allele complementary to rs1434536 polymorphism. Previous genotyping on HEC-1-A cells verified that this cell line contained homozygous reference allele at rs1434536, which is complementary to miR-125b-wt, so transfecting cells with miR-125b-mt would mimic the disrupted binding via BMPR1B 3′UTR with variant allele of rs1434536. Cells transfected with empty vector expressing GFP served as control, which had about 30% growth after 96 hours with G418-containing selection medium. Meanwhile, cells transfected with miR-125b-wt had much less cell death and proliferated almost twice as fast as controls (Fig. 3B). As expected, cells transfected with miR-125b-mt with mutation at complementary position of rs1434536 almost completely eradicated miR-125b effect on endometrium cells. As with proliferative diseases like cancer, endometrium cells are highly migratory. BMPs have been previously shown as potent stimulators of cell migration in endothelial cells [34], making us curious about BMPR1B's role in migration. As shown in Fig. 3C, in cells transfected with control vector or miR-125b-mt, about 4% of total cells migrated to the center region; while with miR-125b-wt transfection, over 6% of total cells migrated. This suggests that migration ability of HEC-1-A cells is greatly enhanced with transfection of miR-125b-wt and significantly attenuated by the miR-125b-mt allele.

A previous miRNA microarray study reported nearly a two-fold increase of miR-125b in ectopic endometrial tissue [35], and reduced BMPR1B expression was also found to promote proliferation of breast cancer cells [36]. Hence, BMPR1B repression by miR-125b may drive proliferation and migration of endometrial cell in ectopic regions. In cell assay, the transfected miR-125b-mt could not overwrite such repression due to endogenous wild-type miR-125b. However, in individuals with homozygous variant allele of rs1434536, the miR-125b binding could be strongly disrupted that may disfavor ectopic cell growth and migration, resulting in prevention of endometriosis progression.

### BMPR1B suppression augments CA125 expression and increases IL-1β expression

We measured mRNA levels of *BMPR1B* in miR-125b-wt transfected cells with regard to miR-125b-mt transfected cells. At 48 hrs post-transfection, positively transfected cells were enriched by fluorescence-activated cell sorting (FACS) to ensure over 90% cells overexpressing miRNA constructs. Quantitative PCR showed that cells transfected with miR-125b-wt yielded 50% reduction of *BMPR1B* transcripts, whereas miR-125b-mt restored BMPR1B levels in endometrial cells (Fig. 3D). This finding confirms that polymorphism in a recognition site diminishes miR-125b-directed repression of endogenous *BMPR1B*, leading to suppression of CA125 overproduction (Fig. 3D).

In order to search for the regulatory network linking BMP signaling pathway and CA125 expression, we quantified CA125 levels and examined several mediator candidates: SMAD1, glucocorticoid receptor (GCCR) and interleukin-1beta (IL-1β) in mi-RNA treated cells. As a well-known downstream effecter of BMP pathway, SMAD1 showed no significant change with overexpression of miR-125b-wt or miR-125b-mt (Fig. 3C), implying modification of its phosphorylation state rather than protein level as the major response to altered BMP signaling. An earlier study suggested glucocorticoid as an inhibitor of CA125 expression [37], while BMP-2 was recently shown to up-regulate GCCR [38]. We thus postulated that BMPR1B suppression may trigger CA125 expression by reducing GCCR activity. Consistently, GCCR levels exhibited a variation slightly below our significance cutoff (*P* = 0.064) in cells transfected with miR-125b-mt construct. About 60% expressional reduction was noted in cells overexpressing miR-125b-wt, and about 30% reduction in cells overexpressing miR-125b-mt versus controls (Fig. 3D).

On the other hand, Q-PCR data revealed more than two-fold increase of IL-1β mRNA level in cells overexpressing miR-125b-wt versus controls or those expressing miR-125b-mt (Fig. 3D). In previous studies, BMP was reported as a regulator for IL-1β expression [39], a cytokine linked to chronic inflammation [40]. IL-1β can enhance endometrium cell adhesion to extracellular matrix and promote angiogenesis, contributing to malignant transformation [41], [42]. Studies on ectopic endometrial tissues by using quantitative PCR revealed that higher levels of IL-1β could be found in patients than in controls (Fig. 3E; *P* = 0.021), which is consistent with the findings in previous studies [43], [44]. Therefore, cytokines like IL-1β may be key players that convey physiological outcome of BMP pathway and modulate CA125 expression or release.

## Discussion

**Figure 4:**
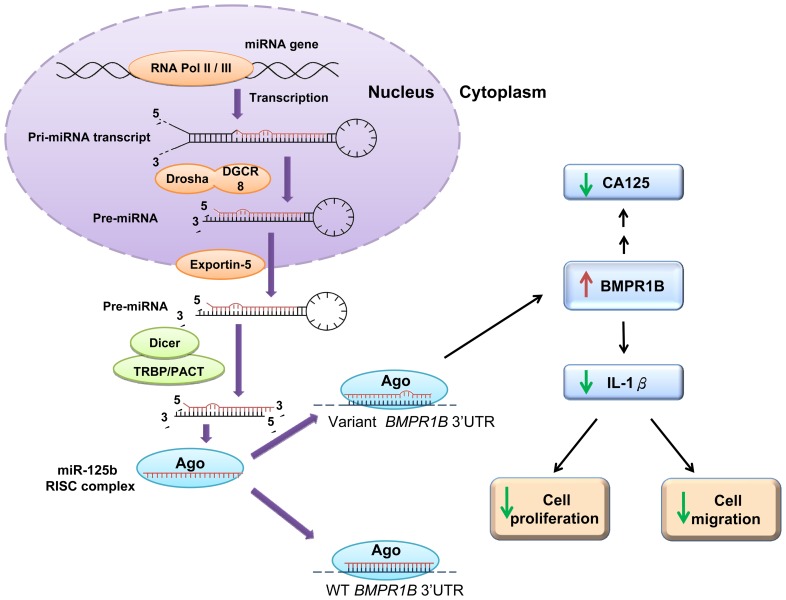
Schematic representation of a proposed model depicting functional influences of genetic variations in *BMPR1B* 3′UTR on endometriosis development. Mature miRNAs including miR-125b are produced by several processing steps where the microprocessor complex (Drosha/DGCR8), exportin 5 and Dicer/TRBP/PACT complex have been known to involve in. The resulting miR-125b forms a RISC complex with Ago and silences BMPR1B gene by binding to the complementary sequence in *BMPR1B* 3′UTR. Impaired recognition due to genetic variations in the mir-125b seed region reduces suppressive effect of mir-125b, resulting in up-regulation of BMPR1B. The CA125 level, a biomarker for endometriosis and ovarian cancer, was found reversely correlated with BMPR1B in endometrium cells. Elevated BMPR1B levels in endometrium cells have been proven to reduce cell proliferation and migration activity via down-regulation of IL-1β, indicating a lower risk to develop endometriosis.

Our study indicates involvement of epigenetic regulation in endometriosis development via miRNA-facilitated repression of BMPR1B, suggesting therapeutic potential of the BMP signaling pathway in endometriosis. The efficacy of miRNA repression depends on degree of complementary between miRNA and its recognition site within a target [7]. Nine tandem bases are complementary to miR-125b within the *BMPR1B* 3′UTR containing the major allele. Imperfect match between miRNA and target caused by genetic variations at the recognition site may lead to insufficient repression of BMPR1B at translation level. Consistent with tumor suppressive role of BMP pathway, we found that up-regulation of *BMPR1B* gene by disruption of miR-125b binding had protective effect against abnormal cell proliferation, with suppression of CA125 expression and serum release of CA125. On the other hand, patients with the variant allele of rs1434536 could maintain relatively unaffected levels of BMPR1B, CA125, and IL-1β, offsetting elevation of miR-125b level presented in endometrium cells, which may prevent endometriosis (Fig. 4).

It is well known that endometriosis exhibits clinical features analogous to cancer: e.g., high growth rate and mobility of endometrial cells [45]. Prior studies afforded convincing evidence of correlation between endometriosis and clear-cell ovarian carcinoma or endometrioid adenocarcinoma [46], [47]. Still, molecular mechanisms underlying transition from endometriosis to ovarian cancer have not been fully addressed. Several molecular alterations are proposed as inducing factors for malignant transformation of endometriosis: hormones, cytokine level variations, mutations or abnormal expression of tumor suppressor genes *TP53*, *PTEN* and oncogenic *KRAS* [48], [49]. Down-regulation of *BMPR1B* expression was recently reported in malignancies such as epithelial ovarian cancer [27], gliomas [50], and breast cancer [36], correlating with poor prognosis in such patients. Coincidentally, Sonoki *et al.* reported an insertion of pre-miR-125b-1 into the immunoglobulin heavy-chain locus in a patient with leukemia, supporting an oncogenic role of miR-125b [19]. Our study may shed light on molecular pathways driving cell proliferation and migration, which are crucial steps for initiating endometriosis and even related malignancies. Further investigation of the molecular network may reveal etiology of endometriosis and establish a system to evaluate malignant transition propensity of endometriosis based on these molecular markers.

As the most commonly employed tumor marker, CA125/MUC16 level is frequently elevated in endometriosis and more often in ovarian cancer [51]. CA125, a transmembrane glycoprotein, consists of a short intracellular and giant extracellular domain with 22,097 amino acid residues [20]. Unfortunately, extremely large molecular size makes it difficult to clone *CA125/MUC16* (>30 Kb) gene in full-length gene, and the transfection efficiency could be too low for any structural or functional study. Yet CA125 emerged as a direct metastasis-contributing factor via mediating adhesion of ovarian cancer cells to the peritoneal surface [52], [53]. Recent study confirmed that silencing of CA125 substantially subdued adhesion, migration, and invasiveness of ovarian cancer cells [54]. Our immunostaining featured active CA125 production and secretion in patients with homozygous reference allele of rs1434536 (Fig. 2C). This finding implies a potential for CA125 to facilitate cell migration and adhesion toward surrounding tissue during endometriosis development. Though increase of BMPR1B in endometriosis epithelium cells is subtle via variants in the miR-125b binding site, predominant BMPR1B expression in stroma cells seems sufficient to suppress CA125 production by neighboring epithelium cells.

Our luciferase assay confirmed more effective posttranscriptional repression through *BMPR1B* 3'UTR containing the major C allele in endometrium cell lines, as compared to previous assay performed in breast cancer cell lines [28]. Moreover, reduction of *BMPR1B* mRNA level by miR-125b-wt was much stronger compared to the mRNA suppression in breast cancer cells [28], suggesting expressional regulation exerted by miR-125b as more predominant in endometrial tissue, possibly due to tissue-dependent variation of endogenous miRNA levels.

## Conclusions

Our data demonstrate miRNA-directed gene repression can be relieved by a single base variation at the binding site that may protect against endometriosis development. Polymorphism at rs1434536 rescued BMPR1B from miR-125b targeted suppression, reducing abnormal cell proliferation as well as CA125 level. Such epigenetic variation may alter certain cytokine-related signaling networks responsible for progression or malignant transition of endometriosis.

## Supporting Information

Table S1 SNPs in *BMPR1B* and cognate probes used in this study.
(DOC)

Table S2 Primer sequences for quantitative real-time PCR and gene cloning.
(DOC)
