# Supplementary material for: BMPR1B Up-Regulation via a miRNA Binding Site Variation Defines Endometriosis Susceptibility and CA125 Levels
Source: PLoS One. 2013 Dec 5;8(12):e80630. doi: 10.1371/journal.pone.0080630 (PMC3855056; doi:10.1371/journal.pone.0080630)
Supplement: Table S1 — SNPs in BMPR1B and cognate probes used in this study. (DOC) [file pone.0080630.s001.doc]

**Table S1 SNPs in *BMPR1B* and cognate probes used in this study**

| **SNP** | **Allele frequencies*** | | **ABI probe assay ID** | **Chr. location** |
| --- | --- | --- | --- | --- |
| rs1970801 | G:0.57 | T:0.43 | C_2913363_10 | 4: 96070525 |
| rs1434536 | C:0.62 | T:0.38 | C_8933587_10 | 4: 96075965 |
| rs11097457 | A:0.57 | G:0.43 | C_2913355_10 | 4: 96076813 |

*Allele frequencies listed are from Chinese Han in Beijing population data in HapMap database.
