# Supplementary material for: BMPR1B Up-Regulation via a miRNA Binding Site Variation Defines Endometriosis Susceptibility and CA125 Levels
Source: PLoS One. 2013 Dec 5;8(12):e80630. doi: 10.1371/journal.pone.0080630 (PMC3855056; doi:10.1371/journal.pone.0080630)
Supplement: Table S2 — Primer sequences for quantitative real-time PCR and gene cloning. (DOC) [file pone.0080630.s002.doc]

**Table S2** Primer sequences for quantitative real-time PCR and gene cloning.

| Gene | Forward primer | Reverse primer |
| --- | --- | --- |
| *For quantitative real-time PCR* | | |
| BMPR1B | tgatggacctatacaccacagg | atagtcctttggaccagcagag |
| MUC16 (CA125) | tggaccttgggacctcag | gagagggccagcagatgtag |
| SMAD1 | tcccctgccctcagaaat | gcaccagtgttttggttcct |
| GCCR | cttcaaaagagcagtggaaggt | gcatgctgggcagttttt |
| IL-1β | aaagcttggtgatgtctggtc | ggacatggagaacaccacttg |
| GAPDH | agccacatcgctcagacac | gcccaatacgaccaaatcc |
|  |  |  |
| *For gene cloning of 3’-UTR region of BMPR1B* | | |
| Forward primer | gtacgctagcgaggaaaagtaagcatctctgcag | |
| Reverse primer | gcatggatccggagtattctgggaggaatgtct | |
